# Isolation and Characterization of *Ftsz* Genes in Cassava

**DOI:** 10.3390/genes8120391

**Published:** 2017-12-15

**Authors:** Meng-Ting Geng, Yi Min, Yuan Yao, Xia Chen, Jie Fan, Shuai Yuan, Lei Wang, Chong Sun, Fan Zhang, Lu Shang, Yun-Lin Wang, Rui-Mei Li, Shao-Ping Fu, Rui-Jun Duan, Jiao Liu, Xin-Wen Hu, Jian-Chun Guo

**Affiliations:** 1 Institute of Tropical Bioscience and Biotechnology, Chinese Academy of Tropical Agricultural Sciences, Haikou 571101, China; mengtinggeng8908@163.com (M.-T.G.); yaofaraway1@163.com (Y.Y.); liruimei@itbb.org.cn (R.-M.L.); fushaoping@itbb.org.cn (S.-P.F.); duanruijun@itbb.org.cn (R.-J.D.); liujiao@itbb.org.cn (J.L.); 2 Hainan Key Laboratory for Sustainable Utilization of Tropical Bioresource, Institute of Tropical Agriculture and Forestry, Hainan University, Haikou 570228, China; minyi0723@126.com (Y.M.); 15298909742@163.com (X.C.); fanjie910102@163.com (J.F.); 18689606176@163.com (S.Y.); 18489972762@163.com (L.W.); zhangfan7116@163.com (F.Z.); 13948427598@163.com (L.S.); 13876830997@163.com (Y.-L.W.); 3 College of Life Science and Biotechnology, Heilongjiang Bayi Agricultural University, Daqing 163319, China; sun780347812@163.com

**Keywords:** isolation, *FtsZ*, cassava, subcellular localization, gene expression, phytohormone response, plastid division, transgenic *Arabidopsis*

## Abstract

The filamenting temperature-sensitive Z proteins (FtsZs) play an important role in plastid division. In this study, three *FtsZ* genes were isolated from the cassava genome, and named *MeFtsZ1*, *MeFtsZ2-1*, and *MeFtsZ2-2*, respectively. Based on phylogeny, the MeFtsZs were classified into two groups (FtsZ1 and FtsZ2). MeFtsZ1 with a putative signal peptide at N-terminal, has six exons, and is classed to FtsZ1 clade. MeFtsZ2-1 and MeFtsZ2-2 without a putative signal peptide, have seven exons, and are classed to FtsZ2 clade. Subcellular localization found that all the three MeFtsZs could locate in chloroplasts and form a ring in chloroplastids. Structure analysis found that all MeFtsZ proteins contain a conserved guanosine triphosphatase (GTPase) domain in favor of generate contractile force for cassava plastid division. The expression profiles of *MeFtsZ* genes by quantitative reverse transcription-PCR (qRT-PCR) analysis in photosynthetic and non-photosynthetic tissues found that all of the *MeFtsZ* genes had higher expression levels in photosynthetic tissues, especially in younger leaves, and lower expression levels in the non-photosynthetic tissues. During cassava storage root development, the expressions of *MeFtsZ2-1* and *MeFtsZ2-2* were comparatively higher than *MeFtsZ1*. The transformed *Arabidopsis* of *MeFtsZ2-1* and *MeFtsZ2-2* contained abnormally shape, fewer number, and larger volume chloroplasts. Phytohormones were involved in regulating the expressions of *MeFtsZ* genes. Therefore, we deduced that all of the *MeFtsZs* play an important role in chloroplast division, and that *MeFtsZ2* (2-1, 2-2) might be involved in amyloplast division and regulated by phytohormones during cassava storage root development.

## 1. Introduction

Plastids compose a metabolically varying group of organelles, which originated from endosymbiosis of an ancient cyanobacterium [1]. The chloroplast is the most conventional and best studied type of plastid, which is responsible for photosynthesis in all photosynthetic eukaryotes [2]. In addition, many non-photosynthetic plastids in plants perform other critical metabolic functions. For instance, the amyloplast, as one of the high differentiated plastids, is related to starch synthesis and storage in sink tissues, which is important for starch crops, such as cassava and potato. Starch granules that are synthesized within the amyloplast compartment are affected not only by enzyme activities required for sucrose metabolism and starch synthesis [3], but also by amyloplast division processes. Chloroplast division processes and mechanisms are well researched and understood. Several proteins, including FtsZ (filamenting temperature-sensitive Z) [4,5,6,7], ARC6 (accumulation and replication of chloroplasts 6) [8,9], ARC5 (accumulation and replication of chloroplasts 5) [10], PDV1 (plastid division 1), and PDV2 (plastid division 2) [11] form at least three contractile components in the plastids division site as follows: first assembled FtsZ ring (Z ring), ARC5 ring, and plastid-diving (PD) ring [12]. The first assembled Z ring is formed by FtsZ. The FtsZ protein serves as an essential scaffold to recruit the other plastid division related proteins and to generate contractile force by its guanosine triphosphatase (GTPase) for plastid division. *FtsZ* genes have been found in various species of Viridiplantae, including many plants and algae, and these genes are clustered into two families, named as FtsZ1 and FtsZ2 [6,13,14]. Genetic analysis in *Arabidopsis* has showed that the FtsZ1 and FtsZ2 families have different functions in chloroplast division [15]. Recent studies have shown that the members of the FtsZ2 family have a closer relationship with their cyanobacterial counterparts than those of the FtsZ1 family, and these studies shown that FtsZ1 has a short N-terminal peptide similar to that of bacterial FtsZs. In comparison to FtsZ1, FtsZ2 lacks the N-terminal peptide [2,16]. Previous studies have demonstrated that FtsZ1 was probably evolved by the duplication of FtsZ2 in the evolution of green algae [17].

The maintenance and divergence of the two FtsZ families (FtsZ1 and FtsZ2) in diverse Viridiplantae suggests that they evolved to have distinct and critical functions in different plastid divisions, such as chloroplast division and amyloplast division. The functions of FtsZ families on chloroplast division have been well researched and understood [2,18]. However, the mechanism of amyloplast division has less studied and understood. For instance, it has been previously reported that the transgenic potato resulting from the transformation of the *StFtsZ1* gene under the control of the GBSS promoter has increased StFtsZ1 protein levels with altered pasting properties and phosphate content in tubers, which result in less but larger starch granules [19]. Yun and Kawagoe reported that the starch granules in the endosperm of *arc5* mutant rice (*Oryza sativa*) are smaller in size and have a significantly higher starch gelatinization peak temperature than those in the wild-type rice [20]. Rice FtsZs play important roles in starch granule synthesis [21].

Presently, an important consideration in cassava breeding is to increase yield and starch quality by focusing on the expression and regulation of key enzymes in the starch biosynthesis pathway. Amyloplast is the main sink organelle for starch grain synthesis and stores in cassava, and its proliferation directly affects the amount and accumulation of starch granules [22]. The morphology, size, and number of amyloplasts can be altered by regulating plastid division elements, which probably change the distribution, dispersion, and arrangement of starch granules, thus affecting starch quantity and quality. Most studies on FtsZs have focused on the regulation of chloroplast division [23]. FtsZs have been shown to mediate the division of the starch-storing amyloplasts in potato [19], but the mechanism is not completed understood. For example, many studies have shown that plant hormones participate in development of storage organ and improve crop yields (e.g., Cassava storage root and potato tuber); it is unclear the way in regard to the amyloplast division affected by plant hormones.

In this study, three *MeFtsZs* were cloned from cassava. The phylogenetic and structural analyses of the predicted MeFtsZ proteins were studied. Moreover, the subcellular fate was confirmed through *MeFtsZ* transient expression experiments in tobacco leaves. The expression profiles of *MeFtsZ*s in cassava plant organs and tissues; or during storage roots development were examined using qRT-PCR. The functions of *MeFtsZ2-1* and *MeFtsZ2-2* in plastid division were identified. The expressions of *MeFtsZ*s were investigated in response to phytohormones. These results are the basis for further studies on *MeFtsZ*s and their role on the division of cassava starch-storing amyloplasts.

## 2. Materials and Methods

### 2.1. Plant Gaterials

The SC8 cassava cultivar (*Manihot esculenta* Crantz no. SC8) that was obtained from the Tropical Crops Genetic Resource Institute (TCGRI, Danzhou, China) at the Chinese Academy of Tropical Agricultural Sciences (CATAS, Haikou, China) was planted in a field under natural conditions. For the differential expression analyses of the *MeFtsZ* genes in the cassava plant organs and tissues, the young leaves, mature leaves, stems, fibrous roots, phloems, and xylems of storage roots were collected at 90 days after planting; the male and female flowers were collected at 200 days after planting, and the fruits were collected at 225 days after planting. The differential expression analyses of the *MeFtsZ* genes in the storage organs during storage root development, the samples were collected at 90, 135, 180, 225, and 270 days after planting, and three biological replicates (different plants) were collected for analyses. Tissue sampling of cassava storage root was according to the method of Guerrero [24]. For phytohormone treatments, the cassava seedlings cultured in vitro for 30 days were transferred to fresh water for 14 days, then added phytohormones to fresh water with various concentrations: Indole-3-acetic acid (IAA, 30 mM), gibberellic acid (GA, 30 mM), abscisic acid (ABA, 30 mM), methyl jasmonate (MeJA, 50 mM), and salicylic (SA, 100 mM). The leaves, stems, and roots materials from three biological samples (different plants) were collected at 1, 3, 6, 12, 18 and 24 h, respectively. All materials were immediately frozen in liquid nitrogen and stored at −80 °C.

### 2.2. Cloning of MeFtsZ Genes

The RNAplant Plus reagent (TianGen, Beijing, China) was used to extract the total RNA from cassava, and the RNA PCR kit (AMV) Ver.3.0 and Oligo dT-Adaptor Primer (TaKaRa, Dalian, China) were used for reverse transcription. Based on the BLAST analysis of the cassava genome database (http://www.phytozome.net/cassava), a set of gene-specific primers were designed (Table 1) to isolate the full-length complementary DNAs (cDNAs) of the *MeFtsZ* genes. All of the PCR products were cloned into the pMD-18 T vector (Takara, Dalian, China), and were sequenced (Shanghai Sangon Biological Engineering Technology and Services Co., Ltd., Shanghai, China).

### 2.3. Sequence and Structural Analysis

DNAman 6.0 software (Lynnon Biosoft, Quebec, QC, Canada) was used to analyze the MeFtsZ amino acid sequence. The exon-intron structure of each *MeFtsZs* was determined by comparing the sequence of *MeFtsZ* family genes with the genomic sequence in the cassava genome database (http://www.phytozome.net/cassava). The chromosomal distribution and orientation of cassava FtsZ genes were identified by identifying their chromosomal position that was given in the cassava genome database 6.1 version. The gene schematic structure was drawn with the Gene Structure Display Server (http://gsds.cbi.pku.edu.cn/index.php). The SignalP program (http://www.cbs. dtu.dk/services/SignalP/) was used to deduce the signal peptide sequence of the MeFtsZs, and their subcellular localizations were analyzed using the TargetP 1.1 program (http://www.cbs. dtu.dk/services/TargetP/). The FtsZ plastid division protein (gene accession: KGY36214; *PDB: 4m8i.1*) from *Staphylococcus epidermidis* RP62A was regarded as a template for the sequence analysis of MeFtsZs using the Swiss-Model server (http://swissmodel.expasy.org). The conserved regions were analyzed by Pymol software (Delino Scientific, San Carlos, CA, USA). The evolutionary relationship of MeFts*Zs* was analyzed using the neighbor-joining method of the MEGA5 program according to the deduced amino acids with 1000 bootstrap replicates. The evolutionary distances were determined using the Poisson correction method. For the phylogenetic analysis sequence of the FtsZ family from other plants were used.

### 2.4. Construction of GFP-Fusion Proteins and Subcellular Localization Analysis

The open reading frames of *MeFtsZ1*, *MeFtsZ2-1*, and *MeFtsZ2-2*, excluding the stop codon, were amplified with added KpnI and BamHI (for *MeFtsZ1*) or SalI and BamHI (for *MeFtsZ2-1* and *MeFtsZ2-2*) restriction sites. The resulting fragments were fused to the green fluorescent protein (GFP) behind the Cauliflower mosaic virus (CaMV) 35S promoter in the modified plant expression vector pG1300 (*eGFP:pCAMBIA1300*), yielding MeFtsZ1-GFP, MeFtsZ2-1-GFP and MeFtsZ2-2-GFP. The resulting plasmids were used for transformation of *Agrobacterium tumefaciens* LBA4404 strain by freeze-thaw method. The Agrobacterium cells harboring different constructs were grown overnight in YEP medium with appropriate antibiotic selection at 28 °C, collected by centrifugation (10,000× *g*), and resuspended to an OD 600 (optical density at 600 nm) of 1.0 in infiltration medium at room temperature for 3 h before injection into the leaves of three-week-old tobacco (*Nicotiana benthamiana*) plants. After three days transfection, the infiltrated parts of tobacco leaves were cut and detected the fluorescent signals from GFP using a Olympus FluoView™ FV1000 confocal microscope.

### 2.5. Plant Transformation and Identification

The wild-type *Arabidopsis* Columbia (Col-0) ecotype was used for transformation. Seeds were surface-sterilized and sown on 1/2 MS (Murashige and Skoog) medium, and grown under 16 h/8 h at 22 °C. MeFtsZ2-1-GFP, MeFtsZ2-2-GFP and pG1300 were employed for *Agrobacterium*-mediated *Arabidopsis* transformation by the floral dip method [25]. Transgenic plants were selected with 25 mg/L hygromycin. Seeds were bleach-sterilized, stratified for 3 days in 4 °C, and grown in the light for 10–15 days on plates, then transferred to soil, and grown at 20 °C, 70% humidity, and with 8 h/16 h photoperiod at 110 μmol m^2^ s^−1^. To identify T-DNA (Transfer DNA) insertion lines by PCR method. T4 progenies were used to identify the characteristics of plastids. For transmission electron microscopy, leaf samples from wild-type *Arabidopsis*, and the transgenic plants that transformed with MeFtsZ2-1-GFP and MeFtsZ2-2-GFP vector were collected and prepared for resin semi-thin sections. Sections were viewed in a HT-7700 transmission electron microscope (Hitachi, Tokyo, Japan) at 60 kV. For confocal microscopy, images of chlorophyll autofluorescence and GFP in transgenic plants were acquired using a FV3000 confocal microscope (Olympus, Tokyo, Japan) and the chloroplast number was manually counted for each cell.

### 2.6. Real-Time RT-PCR Analysis

The relative mRNA expressions of *MeFtsZs* were analyzed by quantitative real-time RT-PCR (qRT-PCR) using the qRT-PCR primers shown in Table 1. The qRT-PCR primers for each *MeFtsZ* gene were designed for the region of low sequence similarity to the other genes to ensure their specificity. The reactions were performed in a 384-well plate in a volume of 10 μL containing 4 μL of template cDNA, 0.4 μL of H_2_O, 5 μL of 2× SYBR^®^ Premix Ex Taq II (Tli RNaseH Plus), 0.4 μL of forward and reverse primers (10 μM) and 0.2 of μL ROX Reference Dye (50×) (SYBR green reagents were supplied by Takara, Dalian, China). The PCR thermocycler program was as follows: 1 min at 95 °C for one cycle, 45 cycles of 5 s at 95 °C, and 30 s at 60 °C, and melting curve analysis at 95 °C for 15 s, 60 °C for 15 s, and 95 °C for 15 s. Cassava tubulin gene (Phytozome name: 4.1_007598m.g) mRNA was amplified as an internal control to calculate the relative expression using the 2^−ΔΔ*C*t^ method. Three technical replicates were analyzed for each biological sample.

## 3. Results

### 3.1. Identification and Characterization of the MeFtsZ Genes

**Figure 1:**
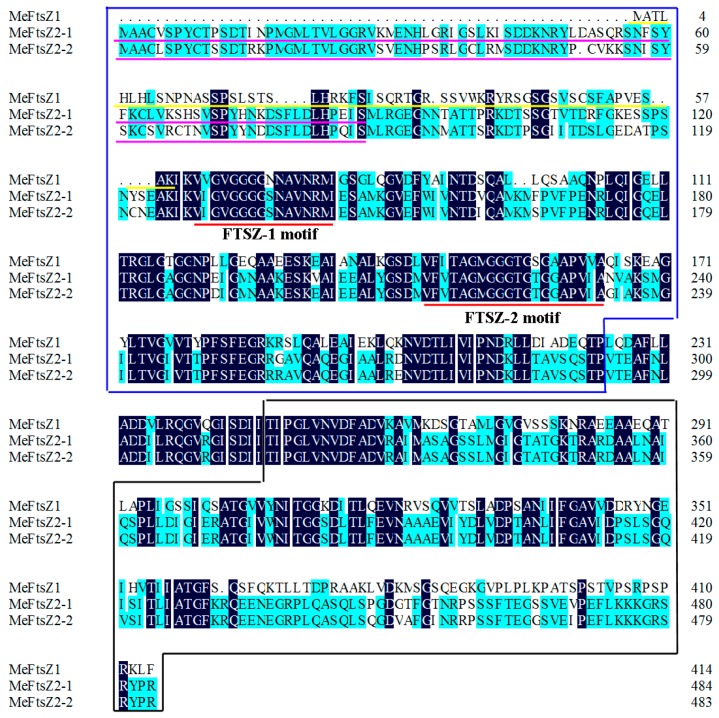
Protein sequence and domain structure of the MeFtsZs. The two conserved sequence domains (FTSZ-1: VIGVGGGGSNAVNRM (PROSITE: PS01134) and FTSZ-2: FATAGMGGT GS/TGAAPV/IV/IA (PROSITE: PS01135) are shown within red lines. The yellow line indicates the putative signal peptide. The pink lines indicate the N-terminal extensions of MeFtsZ2-1 (87 aa) and MeFtsZ2-2 (86 aa), which correspond with chloroplast-targeting proposed by Fujiwara et al. [26]. The N-terminal domain sequence is shown in the blue box, and the C-terminal domain sequence is shown in the black box.

To identify the *FtsZ* genes from cassava, the FtsZ protein sequences from *A. thaliana* and *Ricinus communis* were used as queries in a BLAST to search *MeFtsZ* genes in the publicly available cassava sequences (http://www.phytozome.net/cassava). Three deduced *FtsZ* family genes, named *MeFtsZ1*, *MeFtsZ2-1*, and *MeFtsZ2-2*, were found in the cassava genome. Full-length cDNAs of the three *MeFtsZ* genes were cloned using gene-specific primers by RT-PCR. The cDNA and deduced amino acid sequences of the *MeFtsZs* were deposited in GenBank under the following accession numbers: *MeFtsZ1* (JN936179), *MeFtsZ2-1* (JN936180), and *MeFtsZ2-2* (JQ343216). The open reading frame (ORF) lengths of the three genes of *MeFtsZ1*, *MeFtsZ2-1* and *MeFtsZ2-2* are 1248, 1458 and 1455 base pair (bp), respectively; and their amino acid (aa) sequences range from 415 to 485 aa (Table 2). Alignment analysis of the amino acids shows that the MeFtsZs share 73.61% amino acid identity among the three deduced proteins. MeFtsZ2-1 and MeFtsZ2-2 are found to be highly homologous, sharing 87.84% amino acid identity; while, they have only 46% similar amino acid identity to MeFtsZ1. All of the three MeFtsZs contain two conserved sequence domains (FTSZ-1: VIGVGGGGSNAVNRM (PROSITE: *PS01134*) and FTSZ-2: FATAGMGGTGS/TGAAPV/IV/IA (PROSITE: *PS01135*)). These motifs have been reported to be conserved in the FtsZ genes of green plants (Figure 1). Analysis with the Signal P 4.1 Server indicated that MeFtsZ1 contains a putative signal peptide at the N-terminal amino acids 1 to 58; and, the subcellular localization predicted by the TargetP 1.1 program showed that MeFtsZ1 localizes at chloroplasts. Whereas, MeFtsZ2-1 and MeFtsZ2-2 do not contain putative signal peptide, but contain the N-terminal extensions, which correspond with chloroplast-targeting (Figure 1).

### 3.2. Analysis of the Gene Structure and Chromosomal Distribution of the MeFtsZs

**Figure 2:**
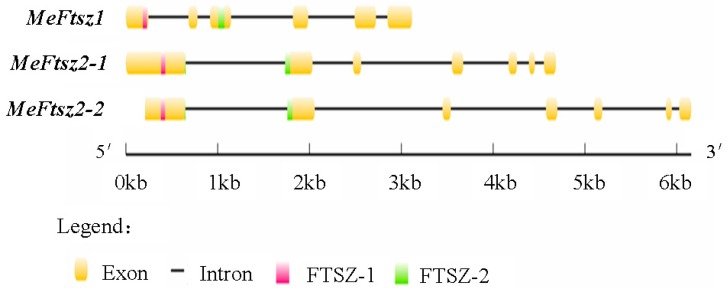
Exon-intron structures of the *MeFtsZ* genes. Introns are shown as black lines, and exons are shown as yellow boxes. FTSZ-1 motif is shown as red boxes, and FTSZ-2 motif is shown as green boxes.

**Figure 3:**
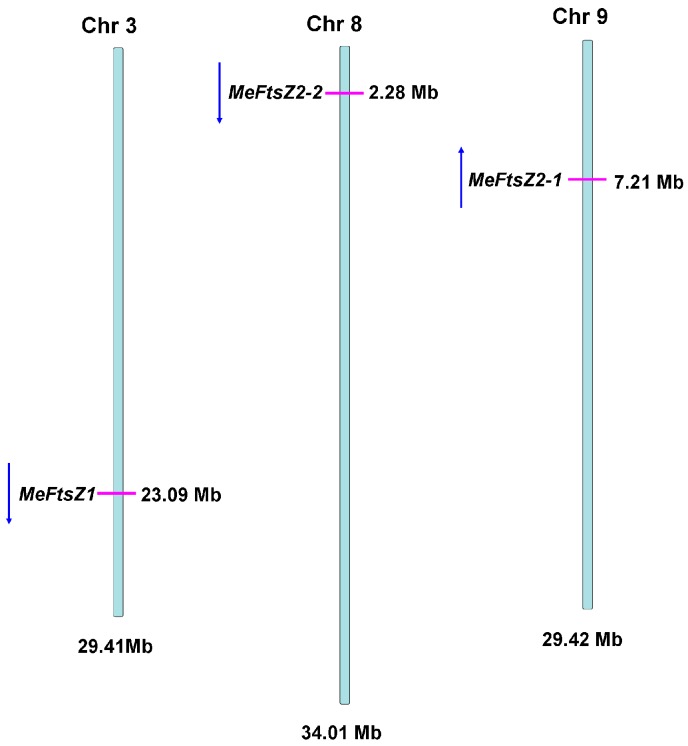
Genome-wide distribution and orientation of *MeFtsZ* genes on cassava chromosomes. Chromosome numbers are shown at the top of each bar. The red lines on the cassava chromosomes indicate the positions of the *MeFtsZ* genes. The blue arrows indicate the gene orientation.

The gene structures of the *MeFtsZ* genes were determined by aligning the cDNA sequences with the genomic sequence from the cassava genome database (http://www.phytozome. net/cassava). The *MeFtsZ* isoforms were found to have two types of exon-intron structures as follows: *MeFtsZ1* has six exons and five introns, while *MeFtsZ2-1* and *MeFtsZ2-2* have seven exons and six introns. The first exon in all of the *MeFtsZs* contains the FTSZ-1, and the FTSZ-2 motifs that locate in different exons (Figure 2). The length of the exons among the *MeFtsZs* was different where the first exon in *MeFtsZ2-1* and *MeFtsZ2-2* was comparatively longer than the others (Figure 2). The three *MeFtsZ* genes were mapped and were found to be distributed in three chromosomes of the cassava genome, in which the *MeFtsZ1* has the same orientation, and locates at chromosome 3; *MeFtsZ2-1* has opposite orientation, and locates at chromosome 9; and, *MeFtsZ2-2* has same orientation, and locates at chromosome 8 (Figure 3).

### 3.3. Phylogenetic Analysis of the MeFtsZ Proteins

**Figure 4:**
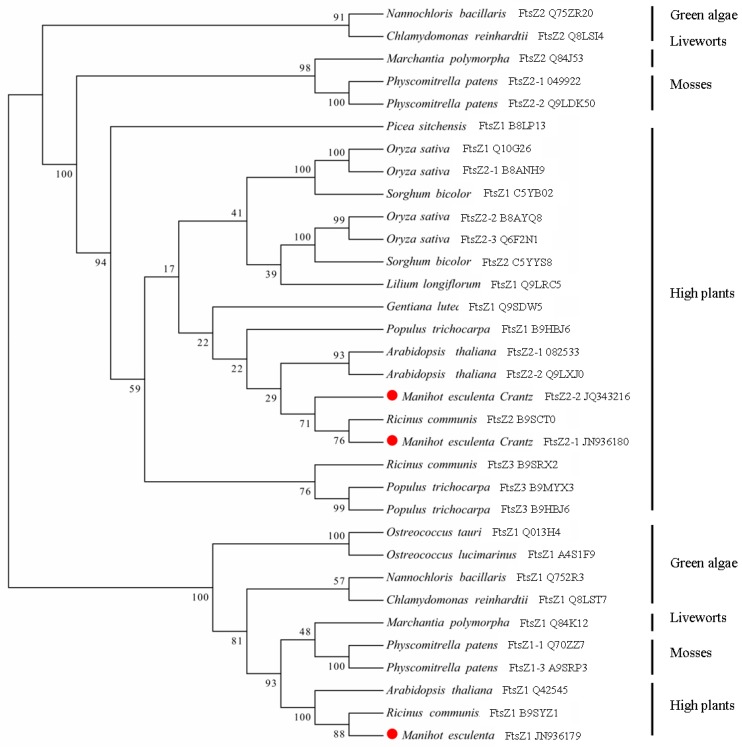
Phylogenetic relationships of the MeFtsZ proteins. The phylogenetic tree was constructed using neighbor-joining algorithm.

The phylogenetic relationship of the MeFtsZs was compared with the FtsZs from the other plants based on the amino acid sequences, using the MEGA5 program. The plant FtsZ sequences were clearly separated into two distinct families (FtsZ1 and FtsZ2) (Figure 4). The MeFtsZs were shown to have a close relationship with the FtsZs from *Ricinus communis*. MeFtsZ2-1 and MeFtsZ2-2 are classified into the FtsZ2 family and have a close relationship with RcFtsZ2 from *R. communis*, sharing 80–82% identity. MeFtsZ1 is classified into the FtsZ1 family, and has a higher homology with RcFtsZ1 from *R. communis*, sharing 85.64% amino acid identity (Figure 4).

### 3.4. Structure Prediction and Homology Modeling of the MeFtsZ Proteins

**Figure 5:**
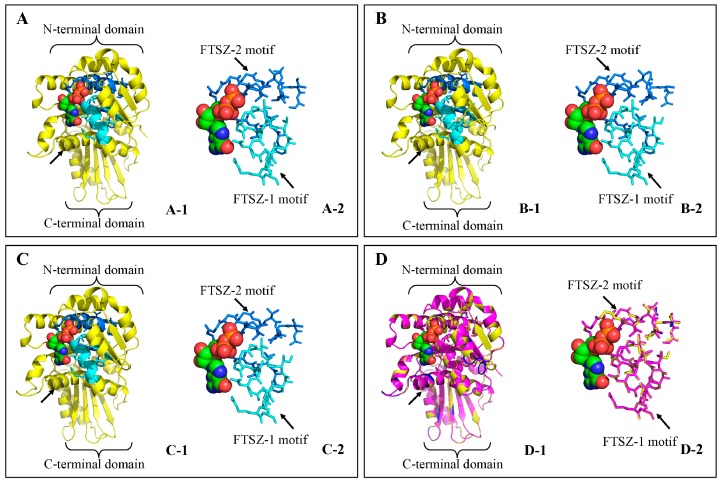
The predicted three-dimensional (3D) structure model of MeFtsZs. (**A**) MeFtsZ1; (**B**) MeFtsZ2-1; (**C**) MeFtsZ2-2; (**D**) comparison of 3D structures from MeFtsZs. A-1, B-1 and C-1 are the predicted 3D structure models of MeFtsZ1, MeFtsZ2-1 and MeFtsZ2-2, respectively. D-1 is the merged 3D structure model of MeFtsZs. Black arrow shows the central α-helix which connects the N-terminal GTPase domain and the C-terminal αβ-domain in the MeFtsZs. The conserved sequence domains (FTSZ-1 motif and FTSZ-2 motif) are shown as sticks in A-2, B-2, C-2 and D-2. The GTP molecule is depicted by colored balls.

To obtain a reasonable theoretical structure of the MeFtsZs, protein homology modeling was performed using a known three-dimensional (3D) structure at 1.43 Å of the FtsZ protein from *Staphylococcus epidermidis* RP62A (*PDB: 4m8i.1*) as a template through the Swiss model server and QMEAN server. The total QMEAN scores (estimated model reliability between 0 and 1) of the predicted 3D models for MeFtsZ1 to 3 were 0.750 (Z-score: −0.27), 0.735 (Z-score: −0.45), and 0.721 (Z-score: −0.60), respectively, which indicated that the three models were reliable. The overall predicted structures showed that MeFtsZ1, MeFtsZ2-1, and MeFtsZ2-2 have similar substrates (Figure 5). The N-terminals and the C-terminal ends of MeFtsZs were found to have a GTPase domain and a αβ-domain, respectively. The α-helix structure connected the N-terminal GTPase domain with the C-terminal αβ-domain in the MeFtsZs. The structures of the FTSZ-1 and FTSZ-2 motifs were closely integrated with the guanosine triphosphate (GTP) molecule, which may allow the MeFtsZ proteins to have GTPase activity.

### 3.5. Subcellular Localization of MeFtsZs

**Figure 6:**
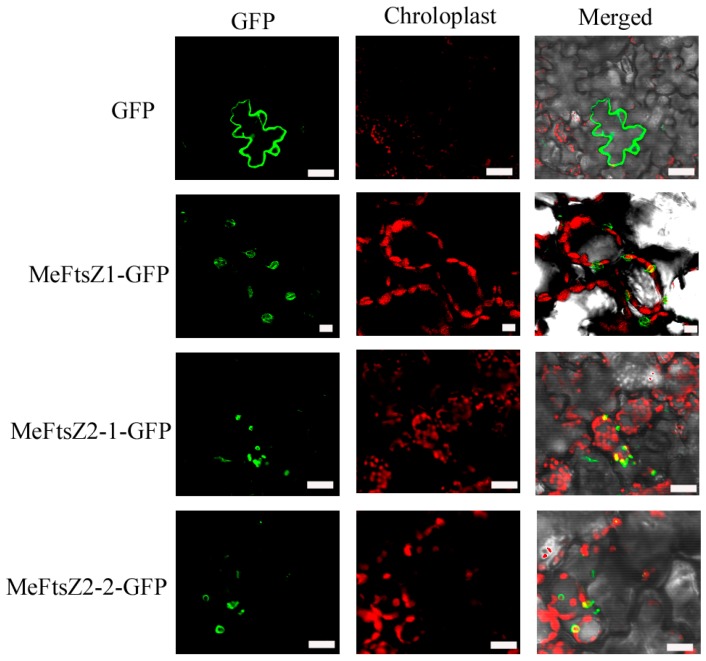
Subcellular localization of the MeFtsZ proteins in tobacco leaf epidermal cells. Green fluorescent protein (GFP) fluorescence is shown in green color, chlorophyll auto fluorescence is shown in red color as a chloroplast marker. Bar = 10 μm.

To study the subcellular localization of MeFtsZs, the coding sequences of the *MeFtsZ* genes were, respectively, fused in-frame with the coding sequence of *GFP*, and then were placed downstream of a cauliflower mosaic virus (CaMV) 35S promoter to be transiently expressed in tobacco leaf epidermal cells. After transfection for three days, the GFP fluorescent could be observed in all chloroplasts of the *MeFtsZ-GFP* transiently expressed tobacco leaves to form a ring in the chloroplasts (Figure 6). Thus, it is suggested that MeFtsZ1, MeFtsZ2-1, and MeFtsZ2-2 function in the plastids of plants. GFP fluorescent of the MeFtsZ1-GFP was not merged with chlorophyll auto fluorescence, while GFP fluorescent of the MeFtsZ2-1-GFP and MeFtsZ2-2-GFP were merged with chlorophyll auto fluorescence to form yellow fluorescence. These differences may indicate that MeFtsZ1 is located in the outside part of the chloroplast, whereas MeFtsZ2-1 and MeFtsZ2-2 are located within the chloroplast stroma.

### 3.6. The Differential Expression Analyses of MeFtsZs in Cassava Organs and Tissues

**Figure 7:**
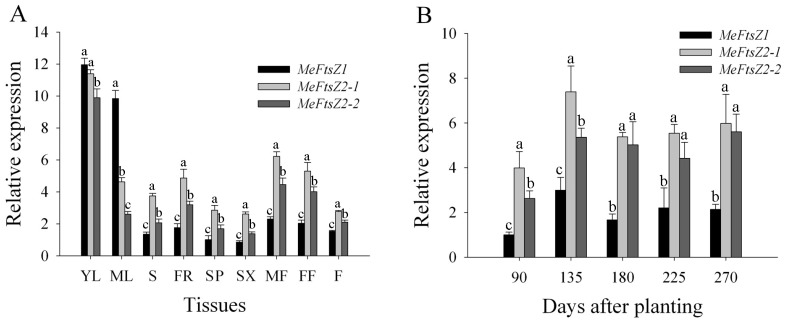
Expression patterns of *MeFtsZ* genes in cassava organs and tissues (**A**), or in storage root xylems during storage root developmental stages (**B**). (**A**) The expression level of *MeFtsZ1* in fibrous roots was used as a calibrator; (**B**) The expression level of *MeFtsZ1* at 90 days was used as a calibrator. The amount of *MeFtsZ*s mRNA was normalized by tubulin mRNA. Each value represents the mean ± SE of three biological replicates (different plants). The abbreviations are as follows: YL, young leaves (the first or second leaves from the top stem); ML, mature leaves (the 7th or 8th leaves from the top stem); S, stems; FR, fibrous roots; SP, storage root phloems; SX, storage root xylems; MF, male flowers; FF, female flowers; and F, fruits. Letters on the error bars indicate the significant difference from each gene by ANOVA analysis (*p* < 0.05).

To determine the expression patterns of the *MeFtsZ* genes in cassava organs and tissues, total RNA was extracted separately from young and mature leaves, stems, fibrous roots, storage root phloems and xylems, male and female flowers, fruits of cassava plants, the mRNAs were isolated, and the cDNAs were synthesized from these organs and tissues for quantitative real-time PCR analysis. The results showed that the *MeFtsZ* genes were expressed in all of the tested tissues. All of the three *MeFtsZs* had the highest expression levels in young leaves. In mature leaves, *MeFtsZ2-1* and *MeFtsZ2-2* reduced their expressions, but *MeFtsZ1* still maintained a high expression level (Figure 7A). In the other organs or tissues, the expression levels of *MeFtsZs* were comparably higher in fibrous roots, male and female flowers; and, the lowest expressions were in storage roots (Figure 7A). The expression of *MeFtsZ2-1* was comparably higher than *MeFtsZ2-2* and *MeFtsZ1* in all of the organs or tissues, except leaves. The expression of *MeFtsZ1* was higher than *MeFtsZ2-1* and *MeFtsZ2-2* in leaves, and the expression of *MeFtsZ1* was comparatively lower than *MeFtsZ2-1* and *MeFtsZ2-2* in all of the non-photosynthetic tissues (Figure 7A).

### 3.7. The Differential Expression Analyses of MeFtsZs in Cassava Storage Root Developmental Stages

To explore the expression of the *MeFtsZ* genes during cassava storage root development, the expressions of the *MeFtsZ* genes were examined by qRT-PCR in the storage root xylems (the location of amyloplast development) of the cassava at 90, 135, 180, 225 and 270 days after planting. The cassava plant initially develops storage roots at 90 days, expands storage roots with starch accumulation at 135 and 180 days, storage root maturity at 225–270 days after planting. All of the three genes had lower expression levels at the storage root initial stage (90 days), and higher expression level at expanding stage (135 and 180 days) and mature stage (225 and 270 days) (Figure 7B). Among the three genes, the expression levels of *MeFtsZ2-1* and *MeFtsZ2-2* were comparatively higher than that of *MeFtsZ1* at all stages (Figure 7B).

### 3.8. The Differential Expression Analyses of MeFtsZ Genes under Phytohormone Treatments

**Figure 8:**
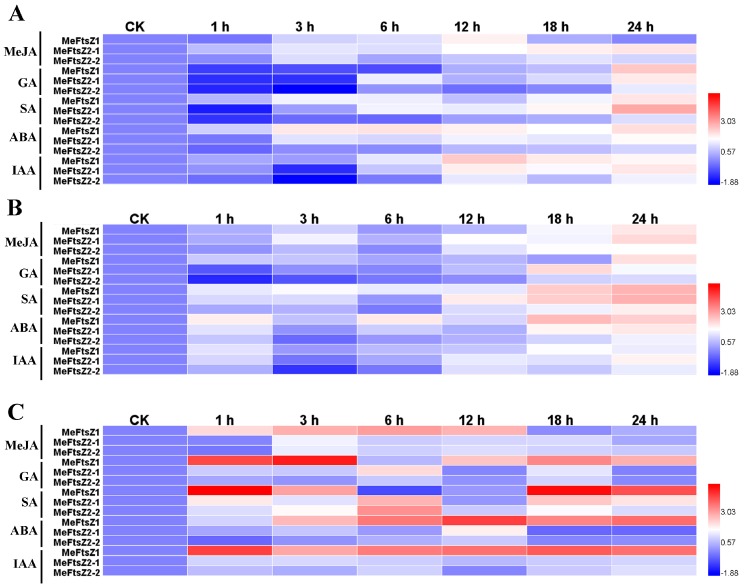
Effects of the phytohormones on *MeFtsZ* gene expressions in leaves (**A**), stems (**B**), and roots (**C**) of cassava seedlings. Cassava seedlings were incubated with 30 μM GA, 30 μM IAA, 100 μM SA, 50 μM MeJA and 30 μM ABA and their leaves, stems, and roots were collected at 1, 3, 6, 12, 18, and 24 h after treatment. Relative expression levels of the *MeFtsZ* genes were analyzed by quantitative reverse transcription-PCR (qRT-PCR), and log2-transformed fold-change values were used for creating the heatmap. Data are means calculated from three biological replicates (different plants). The scale represents the relative signal intensity values.

Phytohormones are involved in the development process of cassava storage roots. In order to investigate the *MeFtsZs* whether to be regulated by phytohormones during cassava storage root development, several phytohormones were used to treat the cassava seedlings; and the expression levels of *MeFtsZ* genes in leaves, stems, and roots were detected. The results showed that all of the *MeFtsZ* genes were responsive to MeJA, GA, SA, ABA, and IAA with variety induced expressing patterns among genes and organs (Figure 8). Under MeJA (50 μM) treatment, *MeFtsZ1* mRNA level was peaked at 12 h in leaves or 24 h in stems; and, peaked at 6 h in roots. The mRNA level of *MeFtsZ2-1*was highly up regulated at later times from 12 h to 24 h, and reached a peak at 24 h in leaves and stems; while, the mRNA levels of *MeFtsZ2-2* had lesser increase than *MeFtsZ2-1*. Both of *MeFtsZ2-1* and *MeFtsZ2-2* were up regulated 3 h in roots. Under GA (30 μM) treatment, *MeFtsZ* genes were down regulated at the initial treatment times (except *MeFtsZ1* was lightly increased in stems), and up regulated at the later treatment times, peaked at 24 h or 18 h (*MeFtsZ2-1* in stems) in the aerial organs of leaves and stems. All *MeFtsZs* were quickly up regulated at 1 h in roots. Under SA (100 μM), *MeFtsZ1* was up regulated from the initial 1 h, and peaked at 24 h in leaves and stems; while, it was highly up regulated at 1 h or 18 h in roots. *MeFtsZ2-1* and *MeFtsZ2-2* were down regulated at the initial treatment times and up regulated at the later times, peaked at 24 h treatment in leaves; all *MeFtsZs* were up regulated and reached a peak at 24 h in stems and were up regulated and reached a peak at 6 h or 12 h in roots. Under ABA (30 μM) treatment, *MeFtsZ* genes were up regulated and reached a peak at 24 h in leaves and stems. *MeFtsZ1* was up regulated a peak at 12 h in roots; while, *MeFtsZ2-1* was up regulated from 1 h to 12 h, then down regulated from 18 h to 24 h; and *MeFtsZ2-2* were down regulated at the initial 1 h, and then up regulated. Under IAA (30 μM) treatment, all *MeFtsZs* were up regulated, and reached a peak at 12 h, or 18 h, or 24 h, respectively, in leaves and stems. *MeFtsZ1* was quickly increased and reached a peak at 1 h in roots; *MeFtsZ2-1* and *MeFtsZ2-2* were down regulated at 3 h, then increased their expressions, and reached a peak at 24 h in leaves and stems; and, they were slightly increased during all of the treated time points in the roots.

### 3.9. Expression of MeFtsZ2-1 and MeFtsZ2-2 in A. thaliana to Identify Their Regulation in Plastid Division

**Figure 9:**
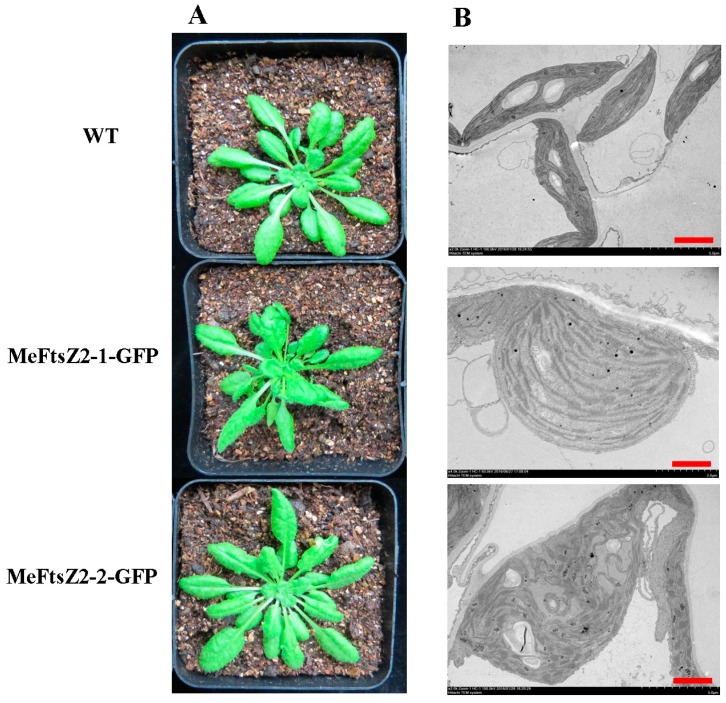
Phenotype of the transgenic and wild type *Arabidopsis* and their mesophyll chloroplasts. (**A**) Phenotype of the wild type and transgenic *Arabidopsis*; (**B**) transmission electron micrographs of the chloroplasts from the wild type and the transgenic *Arabidopsis*. WT, the wild type *Arabidopsis*. MeFtsZ2-1-GFP and MeFtsZ2-2-GFP, the *Arabidopsis* lines were transformed with MeFtsZ2-1-GFP and MeFtsZ2-2-GFP vectors, respectively. Bar = 2.5 μm.

**Figure 10:**
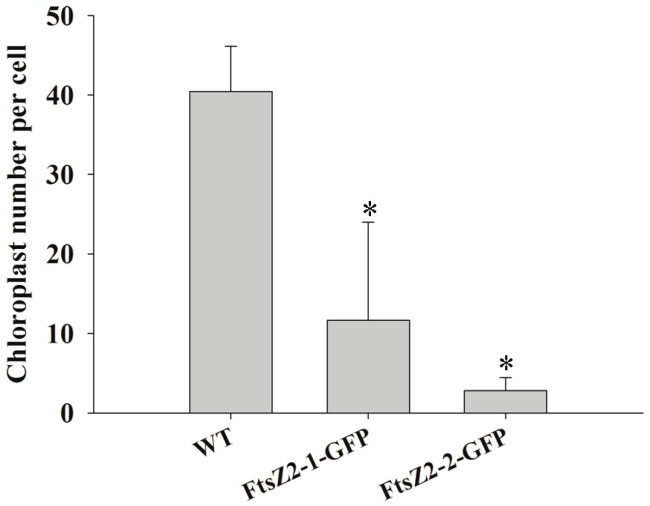
Numbers of the mesophyll chloroplasts in per leaf cell of the transgenic and the wild type *Arabidopsis*. WT, the wild type *Arabidopsis*. MeFtsZ2-1-GFP and MeFtsZ2-2-GFP, the *Arabidopsis* lines were transformed with MeFtsZ2-1-GFP and MeFtsZ2-2-GFP vectors, respectively.

In order to explore the function of *MeFtsZs* in plastid division; two *MeFtsZ* genes of *MeFtsZ2-1* and *MeFtsZ2-2* that have higher expression during storage root development were fused with GFP to transform into the wild-type *A. thaliana* Col-0. The results showed that the phenotype of the transgenic *Arabidopsis* had no obvious changes to the wild type *Arabidopsis* after plant growing in soil for five weeks (Figure 9A); however, the chloroplasts in leaves of the transgenic *Arabidopsis* showed abnormally shape and with large volume under transmission electron micrographs (Figure 9B). The number of chloroplasts in the leaf mesophyll cells of the transgenic *Arabidopsis* was significantly decreased in comparison to the wild type *Arabidopsis*, which was decreased 71.17% and 93.69% in the transgenic lines of MeFtsZ2-1-GFP and MeFtsZ2-1-GFP than in the wild-type *Arabidopsis*, respectively (Figure 10).

**Figure 11:**
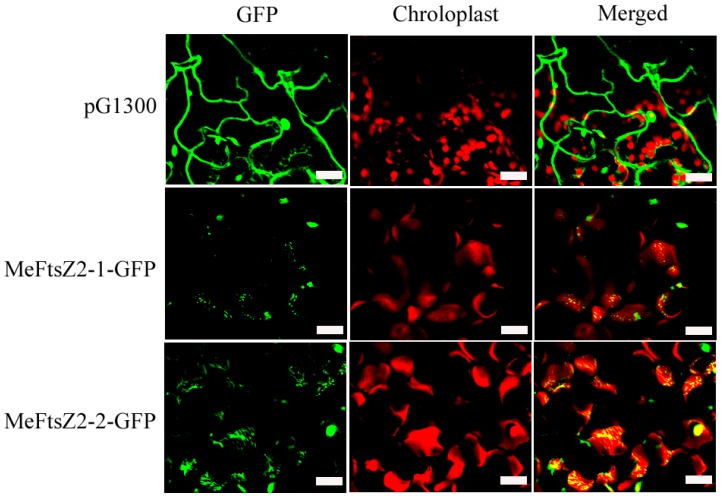
Distribution of MeFtsZ2-1 and MeFtsZ2-1 in mesophyll chloroplasts of the transgenic *Arabidopsis*. WT, the wild type *Arabidopsis*. MeFtsZ2-1-GFP and MeFtsZ2-2-GFP, the *Arabidopsis* lines were transformed with MeFtsZ2-1-GFP and MeFtsZ2-2-GFP vectors, respectively.

In order to determine whether the MeFtsZ2-1 and MeFtsZ2-2 proteins are targeted to the chloroplasts and interfere their division in the transgenic *Arabidopsis*. The GFP fluorescence (green color) and chlorophyll auto fluorescence (red color) in leaf mesophyll cells of the transgenic *Arabidopsis* of MeFtsZ2-1-GFP, MeFtsZ2-2-GFP, and pG1300 (empty vector) were observed under a laser confocal scanning microscope. The results showed that MeFtsZ2-1 and MeFtsZ2-2 were distributed in the chloroplasts of the transgenic *Arabidopsis*, which was showing abnormal morphologies of the dot or filament manners; and an increased size of the chloroplasts was also observed (Figure 11). However, the transgenic *Arabidopsis* with pG1300 vector had normal chloroplast morphology. These results have demonstrated that MeFtsZ2-1 and MeFtsZ2-2 are involved in plant plastid division.

## 4. Discussion

Three full-length cDNAs of MeFtsZs from cassava were cloned in this study. Alignment analysis of the three *MeFtsZ* sequences showed that the two conserved domains (FTSZ-1 and FTSZ-2) reported in the *FtsZ* genes were found in all three of the *MeFtsZ*s [27]. Phylogenetic analysis showed that MeFtsZ2-1 and MeFtsZ2-2 are highly homologous to each other, sharing 87.84% amino acid identity, and with similar genomic structures of seven exons and six introns. However, MeFtsZ1 is lower percentage of sequence similarity to MeFtsZ2-1, MeFtsZ2-2, and with six exons and five introns (Figure 2). The different exon-intron structures have been reported in *AtFtsZs* from *Arabidopsis* [15]. Phylogenetic analysis showed that the MeFtsZ proteins from cassava were grouped to two clades (FtsZ1 and FtsZ2), in which MeFtsZ1 with a prepeptide was classified into FtsZ1 clade; and, MeFtsZ2-1 and MeFtsZ2-2 without a prepeptide were classified into FtsZ2 clade (Figure 3). These results are consistent with the findings in other plants [17,28]. In plants, FtsZs can be divided into two families according to the presence of the plastid guide peptide, FtsZ1 is located in the plastid inner membrane with the presence of a prepeptide, and the FtsZ2 is located in the plastid outer membrane, with the absence of a prepeptide [6]. The localization of FtsZ is closely connected to their function. MeFtsZ1, MeFtsZ2-1, and MeFtsZ2-2 could form a ring in tobacco chloroplasts (Figure 6) suggests that MeFtsZs could be assembled into the “Z ring” and function in the plastid division in cassava plants.

The 3D images of the hypothetical structures of MeFtsZs indicate that all of the three MeFtsZ proteins have an N-terminal GTPase domain and a C-terminal αβ-domain, which are connected by a central α-helix. Furthermore, the two conserved domains, FTSZ-1 and FTSZ-2, are found to be similar among all the three MeFtsZ proteins. The structures of the conserved domains are found to be closely integrated with GTP (Figure 5). The hypothetical structures of MeFtsZs are consistent with the findings of the previous studies [29]. Many studies have shown that the bacterial FtsZs undergo in vitro GTP-dependent assembly into homopolymers. The reaction between the FtsZ subunits forms the GTPase active site, and the polymerization of subunits stimulates GTP hydrolysis. This cycle pattern sustains Z-ring constriction and remodeling [2,30,31]. Similarly, plant FtsZs are capable of GTP-dependent homopolymerization and assembly-stimulated GTPase activity, in vitro [27,32,33,34]. Because, the structures of the cassava MeFtsZs are similar to those in other species, it indicates that they can be assembled into the “Z ring” in the cleavage site of plastid and generate contractile force by the GTPase activity for cassava plastid division.

In Arabidopsis, both deletion and overexpression of *AtFtsZ1* or *AtFtsZ2* cause dose-dependent defects in chloroplast division, and these changes result in a reduction in chloroplast number and an increase in chloroplast size [6,35]. It has been suggested that the expression levels of *AtFtsZ1* or *AtFtsZ2* may be critical for their functions in vivo. Thus, the tissue-specific expression patterns of the *MeFtsZ* genes may provide a basis for understanding their functions during cassava plant development. The expression analysis by qRT-PCR showed that the *MeFtsZ* genes were expressed in all of the tested nine organs or tissues (young and mature leaves, stems, fibrous roots, storage root phloems, storage root xylems, male flower, female flowers, and fruits). The *MeFtsZs* were highly expressed in leaves, especially in younger leaves. Subsequent analysis found that the reduced expression levels of *MeFtsZ2-1* and *MeFtsZ2-2* were found in the mature leaves (Figure 7A). Thus, the *MeFtsZ* genes were highly active in the organs that are rich in chloroplasts, and where chloroplasts more actively divide. In non-photosynthetic tissues, the expression patterns of *MeFtsZ* genes were similar: highest expression of *MeFtsZ2-1*, moderate expression of *MeFtsZ2-2*, and lowest expression of *MeFtsZ1* were detected in these tested non-photosynthetic tissues. These data indicated that the three MeFtsZ proteins are more active in cassava leaves for chloroplast division than in non-photosynthetic tissues for plastid division. Amyloplasts originally developed from one type of plastid, and their divisions cause the accumulation of starch in non-photosynthetic organelles in the sink organs. During the process of storage root development and starch accumulation in cassava, the expression levels of *MeFtsZ2-1* and *MeFtsZ2-2* were higher than that of *MeFtsZ1*, in which *MeFtsZ2-1* and *MeFtsZ2-2* were comparatively more active at the 135 days after planting when cassava storage roots were in the expanding stage where amyloplasts actively divided and accumulated more starch. These data suggested that more MeFtsZ2-1 and MeFtsZ2-2 might be beneficial for amyloplast division during storage root development in cassava.

In order to identify the function of *MeFtsZ2-1* and *MeFtsZ2-2* in plastid division, these two genes were transformed into *A. thaliana*. The results showed that the transgenic plants contained abnormally shape, fewer number, and larger volume chloroplasts (Figure 10 and Figure 11). It has been found that maintaining the balance of plastid division-related gene expression is beneficial to the normal division of plastids [15,19,36]. MeFtsZ2-1 and MeFtsZ2-2 are distributed in the chloroplasts in a dot or a filament manner in order to interfere with the normal chloroplast division in transgenic *Arabidopsis* (Figure 11). Our results demonstrate that MeFtsZ2-1 and MeFtsZ2-2 are involved in the plastid division of plants. However, the mechanisms of *MeFtsZ* genes regulate the divisions of chloroplasts and amyloplasts in the source organs (leaves) and sink organ (storage roots) need further study.

Chromoplasts are plastids that produce and store pigments, such as carotenoid. Raise the content of carotenoid in cassava storage roots will improve nutritional and health benefits of cassava. Analyzed chromoplasts from 23 landraces that have different carotenoid content showed that chromoplast number and size would affect the amount of carotenoids in cassava storage roots [37,38]. Study the expression of *MeFtsZ* genes in different carotenoid content of cassava storage roots may help us to further understand the relationship between chromoplast division and *MeFtsZ* genes.

Phytohormones play the essential roles in cassava storage root initiation and development [39]. The contents of IAA and GA were increased at expanding stage, and then slightly reduced at mature stage in the cassava storage roots, while the content of ABA was gradually increased during the cassava storage root development. Thus, it was clear that IAA, GA, and ABA were involved in the phase transition and development of cassava storage roots [40]. Further, it was found that GA could enhance the mRNA levels of the invertase and sucrose synthase in plants [41]. ABA signaling transduction could regulate the expression of the starch branching enzyme (SBE) gene in cassava storage roots [42]. The addition, of SA and MeJA to culture medium could result in raising the number and weight per shingle plant potato micro tuber [43]. In this study, the expressions of *MeFtsZ* genes were regulated by IAA, GA, ABA, SA, and MeJA; whereas, the expression models of the *MeFtsZ* genes in response to hormone treatment in cassava seedling roots were different. *MeFtsZs* were up regulated and reached a peak after 12 h hormone treatment in aerial organs; *MeFtsZ1* was quickly increased and reached a peak before 6 h in roots. These results indicated that phytohormones were involved in regulating the expressions of *MeFtsZ* genes in aerial organs and storage roots of cassava.

## 5. Conclusions

In conclusion, three *MeFtsZ* cDNAs from cassava were isolated and characterized in this study. The phylogenetic analysis revealed that MeFtsZ2-1 and MeFtsZ2-2 are clustered into the FtsZ2 clade and that MeFtsZ1 is clustered into the FtsZ1 clade. All the three of the MeFtsZs could locate in chloroplasts and form a ring in chloroplastids. All of the *MeFtsZ* genes had higher expression levels in photosynthetic tissues, especially in younger leaves, and lower expression levels in the non-photosynthetic tissues. During cassava storage root development, the expressions of *MeFtsZ2-1* and *MeFtsZ2-2* were comparatively higher than *MeFtsZ1*. The transformed *Arabidopsis* of *MeFtsZ2-1* and *MeFtsZ2-2* contained abnormally shape, fewer number, and larger volume chloroplasts. The higher *MeFtsZ2* (2-1, 2-2) gene expressions might be beneficial for cassava storage root development, during which the phytohormones could play a role through regulating the expressions of *MeFtsZ* genes.

## Figures and Tables

**Table 1:** Gene specific primers used in this study*.*

Primer Name	Sequence (5′ to 3′)	Application
FtsZ1-F	AATGGATCCAAACCCTAAAACCTCT	Gene cloning of *MeFtsZ1*
FtsZ1-R	TGGAAGCTTAAGAAACAACCAACT	
FtsZ2-1-F	CGCGGATCCTACTTCAGCGACTTTAT	Gene cloning of *MeFtsZ2-1*
FtsZ2-1-R	GACGTCGACATGCTCTGTGGAACTATTA	
FtsZ2-2-F	CGCGGATCCTTGACATACTTTTC	Gene cloning of *MeFtsZ2-2*
FtsZ2-2-R	GACGTCGACAACTATTACCTACGG	
FtsZ1-gfp-F	AATGGTACCATGGCGACACTTCATCT	Construction of *MeFtsZ1* fused with *GFP* (green fluorescent protein)
FtsZ1-gfp-R	CACGGATCCAAAGAACAGCTTTCTAG	
FtsZ2-1-gfp-F	TTAGTCGACATGGCAGCTTGTGTGT	Construction of *MeFtsZ2-1* fused with *GFP*
FtsZ2-1-gfp-R	CATGGATCCAAGTCTTGGATAGCGA	
FtsZ2-2-gfp-F	TATGTCGACATGGCAGCCTGTCTGT	Construction of *MeFtsZ2-2* fused with *GFP*
FtsZ2-2-gfp-R	TATGGATCCAGCTCTTGGATAGCGC	
FtsZ1-qPCR-F	GCACCAGTTGTAGCCCAGATA	Expression analysis of *MeFtsZ1*
FtsZ1-qPCR-R	TCCTTCTGGCTGATGATGTTC	
FtsZ2-1-qPCR-F	GAACAACACAGCAACCACTCC	Expression analysis of *MeFtsZ2-1*
FtsZ2-1-qPCR-R	GAAGGGTGTGGATTTTGGAT	
FtsZ2-2-qPCR-F	CCAATCAACCCCAGTAACAGA	Expression analysis of *MeFtsZ2-2*
FtsZ2-2-qPCR-R	GGATTTTGCTGATGTGAGAG	
Tubulin-F	GTGGAGGAACTGGTTCTGGA	Expression analysis of Tubulin gene
Tubulin-R	TGCACTCATCTGCATTCTCC	

**Table 2:** Information for the cassava *MeFtsZ* genes.

Genes	Accession Numbers	ORF Length (bp)	Length (aa)
*MeFtsz1*	JN936179	1248	415
*MeFtsz2-1*	JN936180	1458	485
*MeFtsz2-2*	JQ343216	1455	484
